# Sensory neurons derived from diabetic rats exhibit deficits in functional glycolysis and ATP that are ameliorated by IGF-1

**DOI:** 10.1016/j.molmet.2021.101191

**Published:** 2021-02-13

**Authors:** Mohamad-Reza Aghanoori, Vicky Margulets, Darrell R. Smith, Lorrie A. Kirshenbaum, Daniel Gitler, Paul Fernyhough

**Affiliations:** 1 Division of Neurodegenerative Disorders, St. Boniface Hospital Albrechtsen Research Centre, Winnipeg, MB, Canada; 2 Department of Pharmacology and Therapeutics, University of Manitoba, Winnipeg, MB, Canada; 3 Department of Physiology & Pathophysiology, University of Manitoba, Winnipeg, MB, Canada; 4 Institute of Cardiovascular Sciences, St. Boniface Hospital Albrechtsen Research Centre, Winnipeg, MB, Canada; 5 Department of Physiology and Cell Biology, Faculty of Health Sciences, and Zlotowski Center for Neuroscience, Ben-Gurion University of the Negev, Beer-Sheva, Israel

**Keywords:** ATP biosensors, Axon regeneration, Bioenergetics, Diabetic neuropathy, Mitochondrial respiration, Neurite outgrowth

## Abstract

**Objective:**

The distal dying-back of the longest nerve fibres is a hallmark of diabetic neuropathy, and impaired provision of energy in the form of adenosine triphosphate (ATP) may contribute to this neurodegenerative process. We hypothesised that energy supplementation via glycolysis and/or mitochondrial oxidative phosphorylation is compromised in cultured dorsal root ganglion (DRG) sensory neurons from diabetic rodents, thus contributing to axonal degeneration. Functional analysis of glycolysis and mitochondrial respiration and real-time measurement of ATP levels in live cells were our specific means to test this hypothesis.

**Methods:**

DRG neuron cultures from age-matched control or streptozotocin (STZ)-induced type 1 diabetic rats were used for *in vitro* studies. Three plasmids containing ATP biosensors of varying affinities were transfected into neurons to study endogenous ATP levels in real time. The Seahorse XF analyser was used for glycolysis and mitochondrial respiration measurements.

**Results:**

Fluorescence resonance energy transfer (FRET) efficiency (YFP/CFP ratio) of the ATP biosensors AT1.03 (low affinity) and AT1.03^YEMK^ (medium affinity) were significantly higher than that measured using the ATP-insensitive construct AT1.03^R122/6K^ in both cell bodies and neurites of DRG neurons (p < 0.0001). The ATP level was homogenous along the axons but higher in cell bodies in cultured DRG neurons from both control and diabetic rats. Treatment with oligomycin (an ATP synthase inhibitor in mitochondria) decreased the ATP levels in cultured DRG neurons. Likewise, blockade of glycolysis using 2-deoxy-d-glucose (2-DG: a glucose analogue) reduced ATP levels (p < 0.001). Cultured DRG neurons derived from diabetic rats showed a diminishment of ATP levels (p < 0.01), glycolytic capacity, glycolytic reserve and non-glycolytic acidification. Application of insulin-like growth factor-1 (IGF-1) significantly elevated all the above parameters in DRG neurons from diabetic rats. Oligomycin pre-treatment of DRG neurons, to block oxidative phosphorylation, depleted the glycolytic reserve and lowered basal respiration in sensory neurons derived from control and diabetic rats. Depletion was much higher in sensory neurons from diabetic rats compared to control rats. In addition, an acute increase in glucose concentration, in the presence or absence of oligomycin, elevated parameters of glycolysis by 1.5- to 2-fold while having no impact on mitochondrial respiration.

**Conclusion:**

We provide the first functional evidence for decreased glycolytic capacity in DRG neurons derived from type 1 diabetic rats. IGF-1 protected against the loss of ATP supplies in DRG cell bodies and axons in neurons derived from diabetic rats by augmenting various parameters of glycolysis and mitochondrial respiration.

## Highlights

• There is an energy deficit in the form of ATP in cell bodies and axons of sensory neurons derived from diabetic rats.
• Glycolytic capacity and reserve are depleted in sensory neurons from diabetic rats.
• IGF-1 treatment reverses the energy deficit and glycolysis defect in sensory neurons from diabetic rats.
• Glycolysis is a significant source of energy comprising up to 30% of ATP in adult rat sensory neurons.

## 1 Introduction

Glucose is the major source of energy in the central and peripheral nervous system, with neurons having the highest energy demand [1]. Energy supply is more challenging in the peripheral nervous system, where neurons have longer axons that consume up to 70% of the total energy of the neuron [2]. Approximately 50% of all adenosine triphosphate (ATP) is consumed by the dynamic growth cone in order to support the motility and plasticity in chick embryonic ciliary neurons [3]. In particular, unmyelinated axons are more energetically demanding than myelinated axons, consuming 2- to 10-fold more energy per action potential [4]. Mitochondria are known to concentrate in regions of high metabolic demand, including soma, the nodes of Ranvier and axonal terminals with high synaptic activity in CNS neurons [[5], [6], [7], [8]], and sensory terminal boutons are packed with mitochondria [[9], [10], [11], [12]]. In adult mice, *in vivo* imaging of the saphenous nerve revealed enhancement of mitochondrial anterograde movement along the nerve to dispense energy required for impulse conduction and depolarisation in the axonal tip [13]. Oxidative phosphorylation drives many energy-consuming processes in axons and synapses [14]. However, other processes such as fast axonal transport, a high energy-demanding process in the nerve, are solely dependent on local ATP provided by onsite glyceraldehyde-3-phosphate dehydrogenase (GAPDH; a component of glycolysis) for motor protein transportation [15].

Neurons provide energy to distal axons through mitochondrial positioning and local production of ATP along the nerve and lactate shuttling as a result of anaerobic glycolysis by resident glial cells [16,17]. Thus, mitochondrial dysfunction and any energy deficit can contribute to the pathogenesis of a range of peripheral neuropathies such as Charcot-Marie-Tooth disease (CMT) and diabetic sensorimotor polyneuropathy (DSPN) [[18], [19], [20], [21], [22], [23], [24]]. DSPN is the most common complication of diabetes, affecting more than half of persons with diabetes. Distal dying-back nerve degeneration is a unique characteristic of DSPN, in which metabolism of the peripheral nerve is severely disrupted under chronic diabetic conditions [25,26]. At steady state, mRNA and protein levels of enzymes of glycolysis and tricarboxylic acid (TCA) pathways were elevated in nerves in rodent models of type 1 and type 2 diabetes, most probably a reflection of altered Schwann cell phenotype [[27], [28], [29]]. However, metabolic flux measurements revealed that key pathway intermediates of glycolysis and TCA, such as hexose-6-phosphates, citrate and succinate, were depressed in the sciatic nerve of the *db/db* mouse model of type 2 diabetes [29]. The broad spectrum of enzyme activities of these pathways remains to be determined in DRG and nerves: however, reduced activity of key glycolytic enzymes, hexokinase I and glyceraldehyde-3-phosphate dehydrogenase have been observed in the DRG and retina of type 1 diabetic rats, respectively [30,31]. These data reveal that despite elevated intracellular glucose levels, there was a general energy deficit in the diabetic state in the nervous system, as originally discovered in nerve of type 1 diabetic rats [32]. This aberrant metabolic phenotype could reflect, or contribute to, the observed mitochondrial dysfunction.

The contribution of glycolysis and mitochondrial oxidative phosphorylation to the ATP supply in sensory neurons, and the impact of the diabetic condition remains unclear. ATP biosensors as a tool to measure real-time ATP concentration in sensory neurons can shed light on energy status in the peripheral nerves in normal and disease conditions. We tested the hypothesis that loss of ATP in sensory neurons derived from diabetic rats was a consequence of impaired glycolysis and/or oxidative phosphorylation. We have previously shown that insulin-like growth factor-1 (IGF-1) improves mitochondrial oxygen consumption rate (OCR) through activation of AMP-activated protein kinase (AMPK) and protects against nerve degeneration in experimental diabetic neuropathy [33]. Here, we used ATP biosensors in combination with functional assays of OCR and glycolytic flux to determine the mechanism whereby IGF-1 improves energy supply in cultures derived from type 1 diabetic rats. Our results support a major role for IGF-1 in modulating neuronal metabolism under diabetic conditions.

## 2 Materials and methods

### 2.1 Animals

Male Sprague–Dawley rats were maintained under a 12-h light:dark cycle with free access to diet (5001, LabDiet with fat content of not less than 4.5%, MO, USA). Rats were obtained from our facility at a weight of 201–225 g, and a selected cohort of rats (275–325 g) were made diabetic (non-fasting blood glucose > 19 mmol/l) by a single 90 mg/kg i.p. injection of streptozotocin (STZ) (Sigma, St. Louis, MO, USA). No rats died during the study period and at study end all STZ-injected rats remained hyperglycaemic (non-fasting blood glucose > 19 mmol/l) for at least 3 months. Animal procedures were approved by the University of Manitoba Animal Care Committee and followed Canadian Council of Animal Care (CCAC) rules.

### 2.2 Adult DRG neuron culture and treatments

DRGs were isolated from STZ-diabetic and age-matched control rats and dissociated using previously described methods [34]. The approach has been verified as a useful *in vitro* approach for the study of sensory neuron function and phenotype in the context of diabetes [35]. Neurons were cultured in no-glucose Hams F12 media supplemented with Bottenstein's N2 without insulin (0.1 mg/ml transferrin, 20 nM progesterone, 100 μM putrescine, 30 nM sodium selenite 0.1 mg/ml BSA; all additives were from Sigma, St Louis, MO, USA; culture medium was from Caisson Labs, USA). DRG neurons from control rats were cultured in the presence of 10 mM of d-glucose and DRG neurons derived from STZ-induced diabetic rats with 25 mM of d-glucose unless otherwise specified. No neurotrophins or insulin were added to any DRG cultures. Neuronal cell death was no greater than 5% in all culture conditions.

The following pharmacological treatments were used for *in vitro* experiments: 10 nM of IGF-1 (recombinant human, Preprotech Inc., Rocky Hill, NJ, USA), 50 mM of 2-deoxyglucose (DG) (Sigma, St Louis, MO, USA) and 1 μM of oligomycin (Sigma, St. Louis, MO, USA).

### 2.3 ATP biosensor constructs and transfection

**Supplemental Figure 1:**
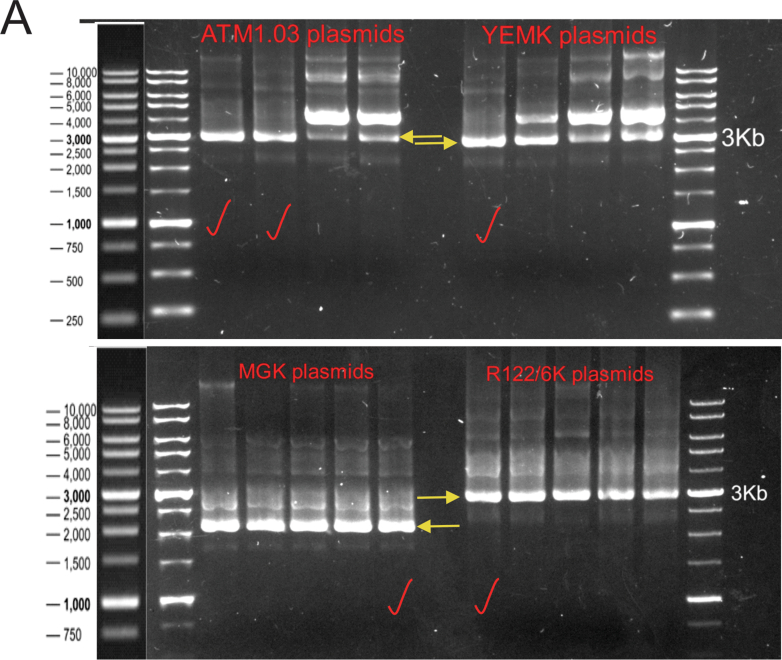
**Validation of size and conformation of plasmids.** Plasmids were run on 0.8% agarose gel. (A) Plasmids (check mark) with the right size and highest level of supercoiled conformation (arrows) were used for transfection and further transformation experiments. Each lane indicates plasmids from one colony of competent DH5α cells carrying an ATP biosensor.

ATP biosensors AT3.10^MGK^ (high affinity), AT1.03^YEMK^ (medium affinity), AT1.03 (low affinity) and AT1.03^R122/6K^ (ATP-insensitive mutant) were originally constructed by Imamura et al. [36]. DH5α-competent cells (New England Biolabs, MA, USA) carrying ATP biosensor constructs were cultured in LB medium at 30 °C overnight. ATP biosensor plasmids were extracted using PureLink™ HiPure Plasmid Filter Maxiprep kit (Invitrogen, California, CA, USA) and ran on 0.8% agarose gel to validate the size and conformation of each plasmid (Supplemental Figure 1A).

Two micrograms of plasmid were used to transfect/electroporate DRG neurons using Amaxa® Rat Neuron Nucleofector kit (Lonza Inc., Basel, Switzerland) according to programme O-003 in an Amaxa nucleofector machine (Lonza Inc., Basel, Switzerland). Neurons were seeded for further experimentation. Transfection efficiency of DRG neurons was 30–40%, which was assessed before imaging and glucose metabolism assays.

### 2.4 Luciferase-based ATP assay

Luminescent ATP detection assay kit (ab113849: Abcam, Cambridge, MA, USA) was used to measure ATP produced by DRG neurons. In brief, cultured DRG neurons and ATP standard dilution series were prepared. D-Luciferin and firefly luciferase reagents were added to the reaction mix, stirred and incubated for 10 min. Luminescence from luciferase activity was recorded using the Glomax multi-detection system (Promega, Wisconsin, USA). A standard curve was plotted and luminescent units from each sample were interpolated to calculate the absolute ATP concentration per mg of total protein lysate. Luminescence from oligomycin- and 2-DG-treated neurons was measured after adjusting to their basal levels.

### 2.5 Glycolysis assay in cultured DRG neurons

For assays of glycolysis, glucose (10 mM; a saturating concentration), oligomycin (1 μM) and 2-deoxy-glucose (2DG: a glucose analogue) (50 mM) were sequentially injected to determine the extracellular acidification rate (ECAR). After ECAR measurement (as part of a glycolysis stress test), parameters such as basal glycolysis rate (under saturating glucose concentration), glycolytic capacity (when oxidative phosphorylation blocked and glycolysis driven to maximum to maintain energy supply), glycolytic reserve (capability of cell to respond to energetic demand; in the absence of a functioning oxidative phosphorylation system) and nonglycolytic acidification were computed following data normalisation to mg protein (DC protein assay, BioRad, USA). Therefore, ECAR measures are presented as mPH/min/mg protein. In the glycolysis assay, (corrected) basal glycolysis was calculated by subtraction of non-glycolytic acidification from raw basal glycolysis when glucose was injected. After inhibition of ATP synthase using oligomycin, (corrected) glycolytic capacity was achieved which was then calculated by subtraction of the non-glycolytic acidification measured from the raw glycolytic capacity. Glycolytic reserve was calculated by subtraction of (corrected) basal glycolysis from (corrected) glycolytic capacity. Basal acidification measures were the ECAR measured before addition of glucose for the assay (comprising all sources of protons in the basal condition), and non-glycolytic acidification was the ECAR measured after addition of ATP synthase inhibitor and glucose analogue (2DG) (comprising all sources of protons except glycolysis and the mitochondrial electron transport chain). Glycolysis accounts for the majority of the ECAR measure [37], and the TCA cycle, glycogenolysis, pyruvate dehydrogenase and glucose-6 phosphate dehydrogenase are the major sources of protons contributing to non-glycolytic acidification [37,38].

### 2.6 Mitochondrial respiration in cultured DRG neurons

Mitochondrial oxygen consumption rate (OCR) was measured in live sensory neurons using the XF24 analyser (Seahorse Biosciences, Billerica, MA, USA). In brief, DRG neurons were isolated from rats and cultured overnight in F12 medium containing 10 mM of d-glucose for cultures derived from age-matched control or 25 mM of d-glucose for diabetic rats. DRG culture medium was changed 1 h before the assay to unbuffered DMEM (Dulbecco's modified Eagle's medium, pH 7.4). The read-outs from OCR measurements were recorded alongside the glycolysis assays with the same injections. Basal mitochondrial respiration was intended before and after injection of glucose and oligomycin and, therefore, calculated for culture groups. Corrected basal respiration was achieved by subtracting non-mitochondrial OCR from raw basal respiration. To calculate corrected basal respiration, rotenone (1 μM) + antimycin A (1 μM) were injected at the end to completely block mitochondrial electron transport. Oligomycin acts as an irreversible ATP synthase inhibitor, rotenone as Complex I inhibitor and antimycin A as an inhibitor of Complex III of the mitochondrial electron transport system. After OCR measurement, basal respiration and non-mitochondrial oxygen consumption were computed following normalization to mg protein (DC protein assay, BioRad, USA). Therefore, OCR measures are presented as pmoles/min/mg protein. Nonmitochondrial respiration measures were OCR measures (oxygen consumed by *e.g.,* peroxisomes) after the addition of inhibitors of Complexes I and III and full blockade of electron transport in mitochondria.

### 2.7 Live cell imaging

For live cell imaging, DRG neurons were cultured on a glass-bottom 2- or 4-chamber culture dish to collect transmitted light with highest efficiency. Live cells were imaged using a spinning disc confocal microscope (Carl Zeiss AG, Oberkochen, Germany) with the following configurations: CFP: Ex 408, Em 475/15, T:0.5 s; YFP: Ex 488, Em 525/15, T:0.025 s and FRET YFP: Ex 408, Em 525/15, T:0.5 s. The microscope was equipped with a 37 °C incubator and connected to a continuous supply of 5% carbon dioxide to maintain cells in physiologic condition while imaging. Fluorescence emission from ATP biosensors was captured using a cooled charge-coupled device (CCD) camera. FRET efficiency (FRET YFP/CFP ratio) was plotted using ROIs calculated pixel-by-pixel in ImageJ. Images were captured after 48 h of culture, to permit stable expression of the biosensors and efficient ATP binding. ATP levels detected were constant in the cell body and axon over the period of 24–48 h (data not shown). All images were captured at sub-saturating fluorescence intensity to allow accurate quantification. Cell bodies and axonal signals were acquired under differing exposures.

### 2.8 Statistical analysis

Data were analysed using two-tailed Student's t-tests or one-way analysis of variance (ANOVA) followed by Tukey's or Dunnett's *post hoc* tests, as appropriate and indicated (GraphPad Prism 7, GraphPad Software). A P value < 0.05 was considered significant.

## 3 Results

### 3.1 Energy deficit in cell bodies and axons of sensory neurons derived from diabetic rats was prevented by IGF-1

**Figure 1:**
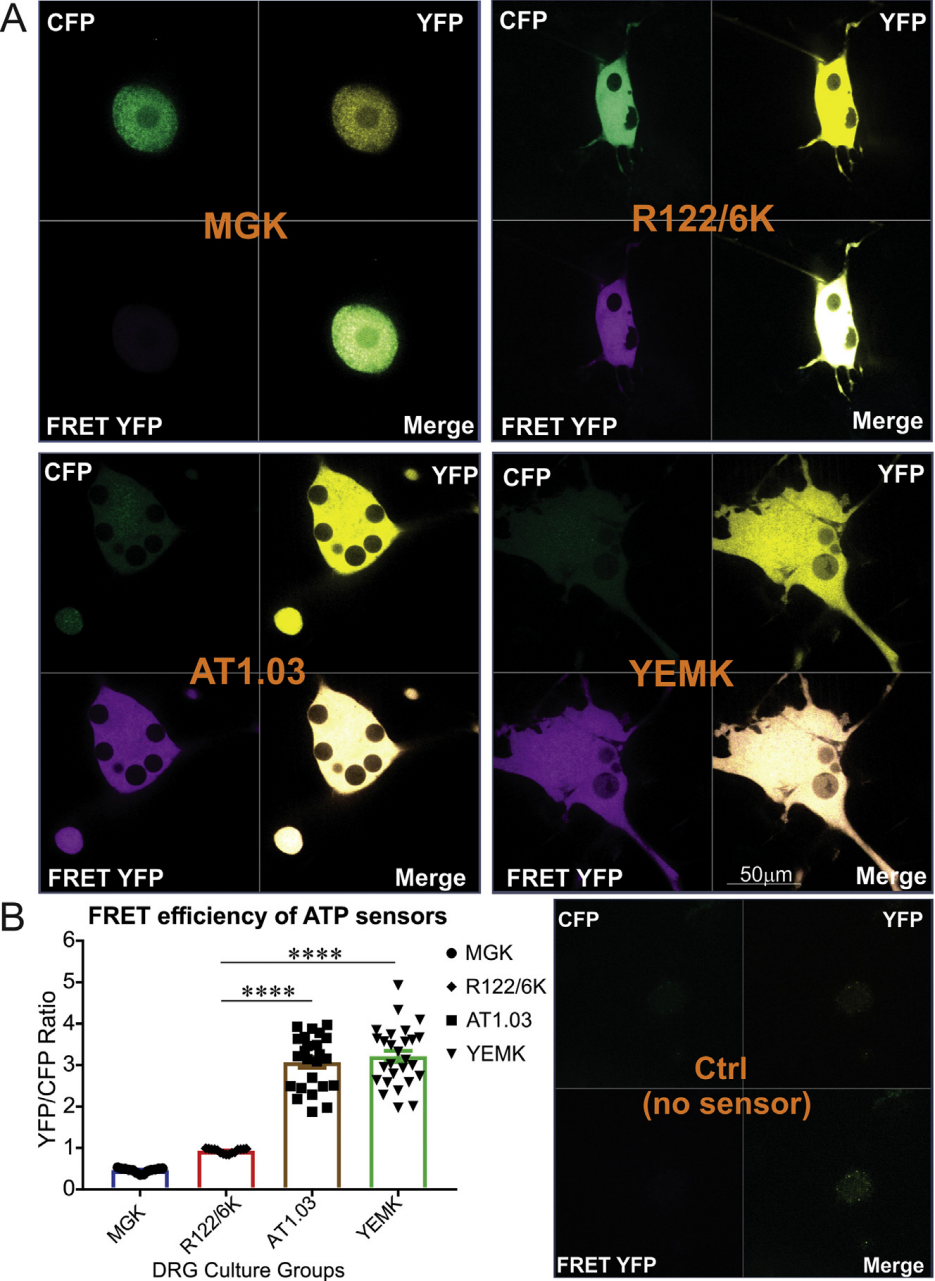
**Wild-type and mutant ATP sensors were detected in sensory neurons derived from rats.** DRG neurons derived from adult control rats were transfected with AT3.10^MGK^ (high affinity), AT1.03^YEMK^ (medium affinity), AT1.03 (low affinity) and AT1.03^R122/6K^ (mutant) constructs for 48 h. Images were taken using confocal microscope and FRET efficiency (YFP/CFP ratio) was calculated for each group of ATP sensors. No FRET signal was detected in MGK-transfected sensory neurons in our *in vitro* system. (A) shows representative fluorescent images and (B) reveals tabulated data, and all groups were compared to mutant as the control group. Data are mean ± SEM of N = 25–35 images (1–3 neurons per image were analysed); ∗∗∗∗ = p < 0.0001; analysed by one-way ANOVA with Dunnett's post-hoc test.

**Supplemental Figure 2:**
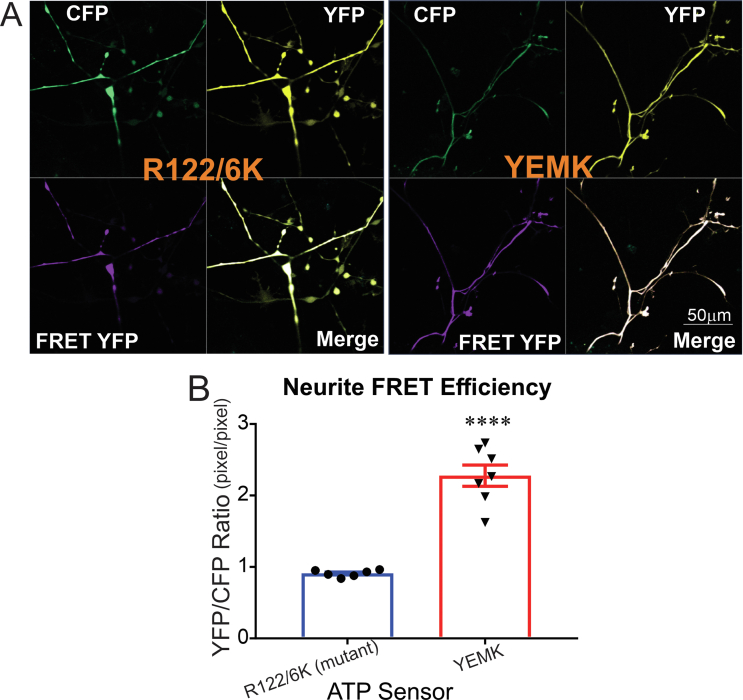
**The higher FRET efficiency of medium affinity construct (AT1.03**^**YEMK**^**) versus mutant construct (AT1.03**^**R122/6K**^**) was also evident in the neurites of cultured DRG neurons.** DRG neurons derived from adult control rats were transfected with AT1.03^YEMK^ (medium affinity) and AT1.03^R122/6K^ (mutant) constructs for 48 h. Images were taken from longest neurite regions using confocal microscope, and FRET efficiency (YFP/CFP ratio) was calculated for each group of cultured neurons expressing a specific ATP sensor. Data are mean ± SEM of N=6-7 neurites from 6-7 different images; ∗∗∗∗=p<0.0001; analysed by Student’s t-test.

To explore the efficiency of each ATP biosensor in cultured DRG neurons derived from adult control rats, the neurons were transfected with AT3.10^MGK^ (high affinity), AT1.03^YEMK^ (medium affinity), AT1.03 (low affinity) and AT1.03^R122/6K^ (ATP-insensitive mutant) constructs and FRET efficiency (YFP/CFP ratio) of each was calculated. In neuronal cell bodies a three-fold higher efficiency was observed in AT1.03^YEMK^- and AT1.03-expressing DRG neurons compared to AT1.03^R122/6K^-expressing neurons (P < 0.0001) (Figure 1). Similar results were observed in neurites (Supplemental Figures 2A and 2B). There was no FRET signal detected in AT3.01^MGK^-expressing neurons which was probably due to a mutation or miss-folding of the Epsilon subunit of the ATP biosensor.

**Figure 2:**
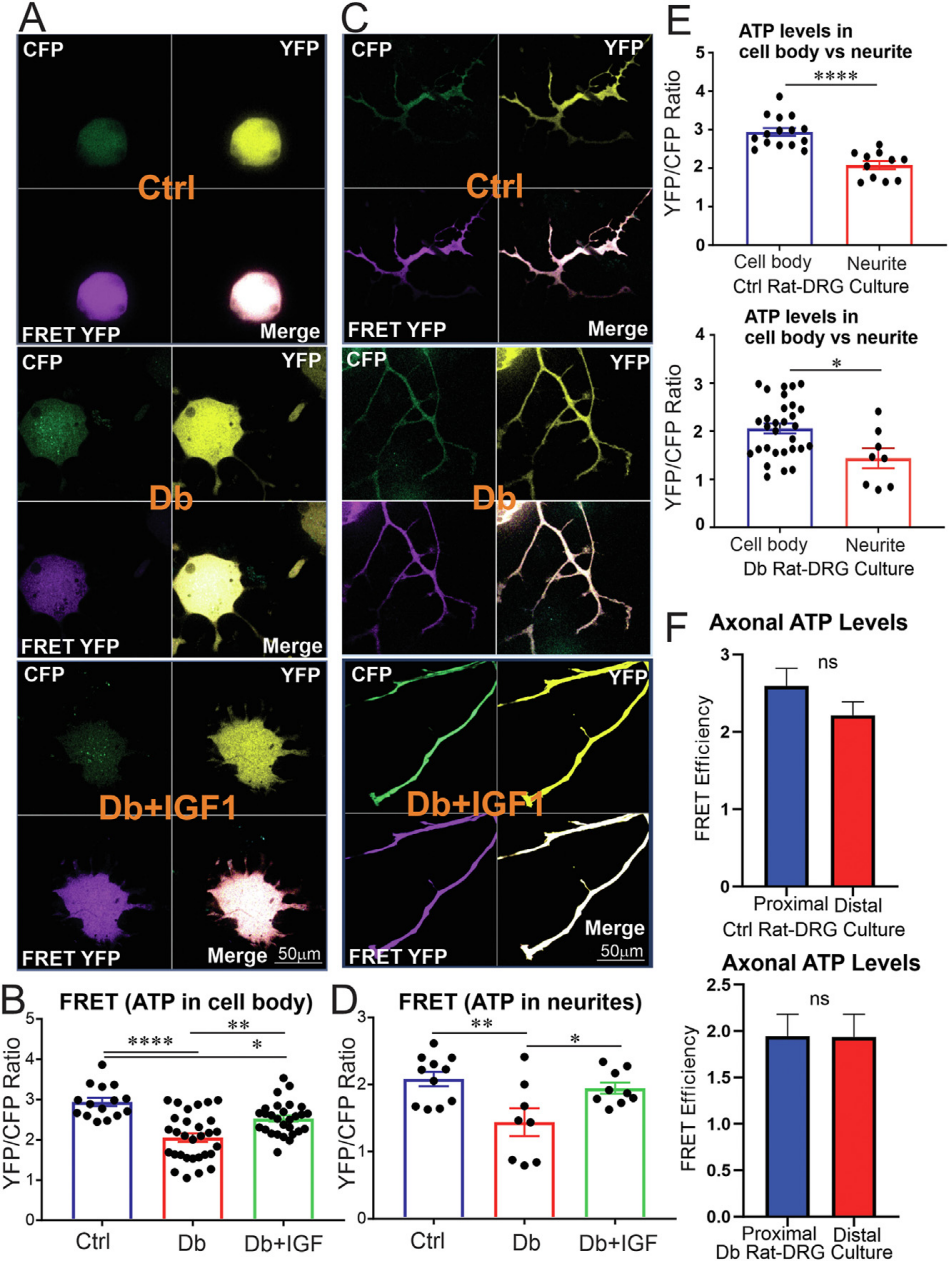
**Energy deficit in sensory neurons from diabetic rats was prevented by IGF-1 treatment.** DRG neurons derived from adult control or STZ-induced diabetic rats were transfected with AT1.03^YEMK^ (medium affinity) for 48 h. A subgroup of DRG neurons from diabetic rats were treated with 10 nM IGF-1 for the final 24 h. (A, B) Cell body and (C, D) axonal images were taken using confocal microscope and FRET efficiency (YFP/CFP ratio) was calculated for each group of ATP sensors. In (E), FRET efficiency of cell body and neurites of the neurons are compared within groups. In (F), FRET efficiency of proximal and distal parts of the longest neurites are compared within groups. Data are mean ± SEM of N > 30 images for cell bodies and N > 8 for axons (1–2 cell bodies/neurites per image were analysed); ∗ = p < 0.05 or ∗∗ = p < 0.01 or ∗∗∗∗ = p < 0.0001; analysed by Student's t-test or one-way ANOVA with Tukey's post-hoc test.

**Supplemental Figure 3:**
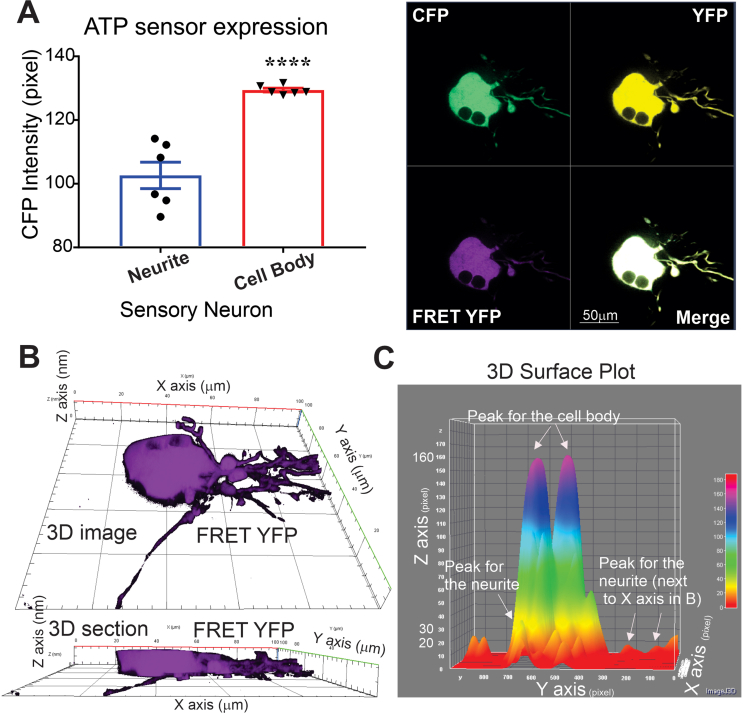
**The depth and intensity of FRET signal is higher in cell bodies than in axons of sensory neurons.** DRG neurons derived from adult control rats were transfected with AT1.03^YEMK^ (medium affinity) for 48 h. Images were taken from cell body and axonal regions using confocal microscope, and FRET YFP as well as CFP intensity were calculated and analysed independently. In (A), CFP intensity was measured in 6 cell bodies and corresponding neurites of the same neurons derived from control rats. A representative image is given. In (B), FRET YFP intensity from the representative image was plotted on X, Y and Z axes for volumetric analysis of neurites and cell body. In (C), color-coded 3D surface plot of the same neuron was made using FRET YFP intensity measures by using ImageJ. Colours represent the depth of the Z axis (in pixel) in the cell body and neurites. Data are mean ± SEM of N=6 images; ∗∗∗∗=p<0.0001; analysed by Student’s t-test.

Considering the higher FRET signal of the AT1.03^YEMK^ compared with the AT1.03 ATP biosensor, we employed AT1.03^YEMK^ for the next experiments. We used the AT1.03^YEMK^ (medium affinity) construct to measure real-time ATP production in cultures of DRG neurons derived from adult control or STZ-induced diabetic rats over a 48h period. A subgroup of cultured DRG neurons derived from diabetic rats were treated with 10 nM of IGF-1 for the final 24 h. At this IGF-1 concentration, signalling is only mediated via the IGF type 1 receptor, with no cross-occupancy of insulin receptors [39]. There was a significantly lower ATP level in cell bodies of DRG neurons and the longest axons from diabetic rats compared to that of control rats (P < 0.001 and P < 0.01, respectively) (Figure 2A–D). Treatment with IGF-1 peptide restored the ATP level in both cell bodies and the longest neurites of DRG neurons from diabetic rats (Figure 2A–D). The ATP level was significantly higher in cell bodies of DRG neurons from control and diabetic rats than in neurites within the same culture group (Figure 2E). Of note, autofluorescence levels across conditions were comparable. No significant difference was found in FRET efficiency between the proximal and distal parts of the longest neurite within culture groups (Figure 2F). In an independent assessment of components of FRET, CFP and FRET YFP, we found that there was 1.3-fold higher CFP intensity in cell bodies of DRG neurons compared to neurites (P < 0.0001) (Supplemental Figure 3A). However, the three-dimensional (3D) image, section and surface plot of FRET YFP in DRG neurons showed 6- to 8-fold higher depth in the cell body compared with the neurite (Supplemental Figure 3B-C). These data suggest that the ATP concentration (FRET efficiency) was higher in the cell body than neurites of cultured DRG neurons confirming Figure 2E (Supplemental Figure 3A-C).

### 3.2 Glycolytic capacity and reserve were depleted in sensory neurons from diabetic rats and enhanced by IGF-1 treatment

**Figure 3:**
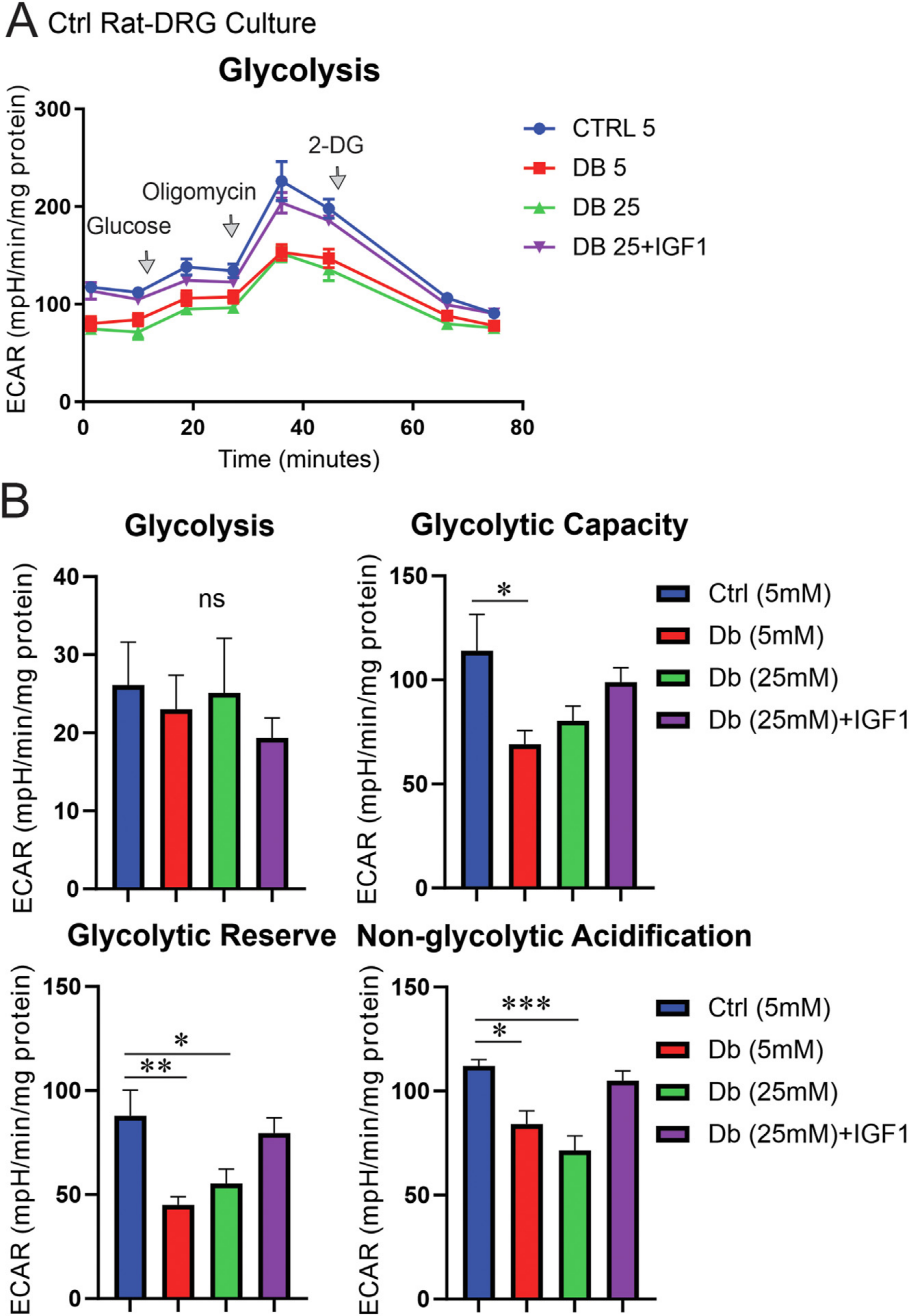
**Glycolytic capacity and reserve were defective in sensory neurons from diabetic rats and were restored with IGF-1 treatment.** DRG neurons derived from adult control and diabetic rats were cultured in the presence of 10 mM or 25 mM of glucose overnight and underwent glycolysis analysis test using the Seahorse XF24 bioanalyser. Media was replaced with no-glucose media 1 h prior to the experiment. Glucose (10 mM), oligomycin (1 μM) and 2-deoxy-glucose (2DG: a glucose analogue) (50 mM) were sequentially injected to determine the extracellular acidification rate (ECAR). More details on glycolysis parameters are given in the method section. Data are mean ± SEM of N = 4–5 replicates; ∗ = p < 0.05 or ∗∗ = p < 0.01 or ∗∗∗ = p < 0.001; analysed by one-way ANOVA with Dunnett's post-hoc test.

To investigate whether the deficit in ATP levels was due to depressed glycolysis in DRG neurons from diabetic rats, DRG neurons derived from adult control and diabetic rats were cultured in the presence of 5 mM or 25 mM of glucose, respectively, and treated with/without 10 nM of IGF-1 for 24 h. The glycolysis analysis test, derived from ECAR measurements, revealed a significant decrease (P < 0.05) in non-glycolytic acidification, glycolytic capacity and glycolytic reserve in DRG neurons derived from diabetic rats compared to DRG neurons from control rats (Figure 3A–B). Of note, diabetic neurons cultured for 24 h in normal glucose (5 mM) still revealed deficits in these parameters of glycolysis, indicating that the aberrant bioenergetic phenotype was not readily reversed under normoglycemia. However, there was no difference in basal glycolysis between culture groups. IGF-1 treatment in DRG neurons from diabetic rats elevated glycolytic capacity, glycolytic reserve and non-glycolytic acidification to normal levels (Figure 3A–B).

### 3.3 Glycolysis is a significant source of ATP in sensory neurons; impairment in diabetes

**Figure 4:**
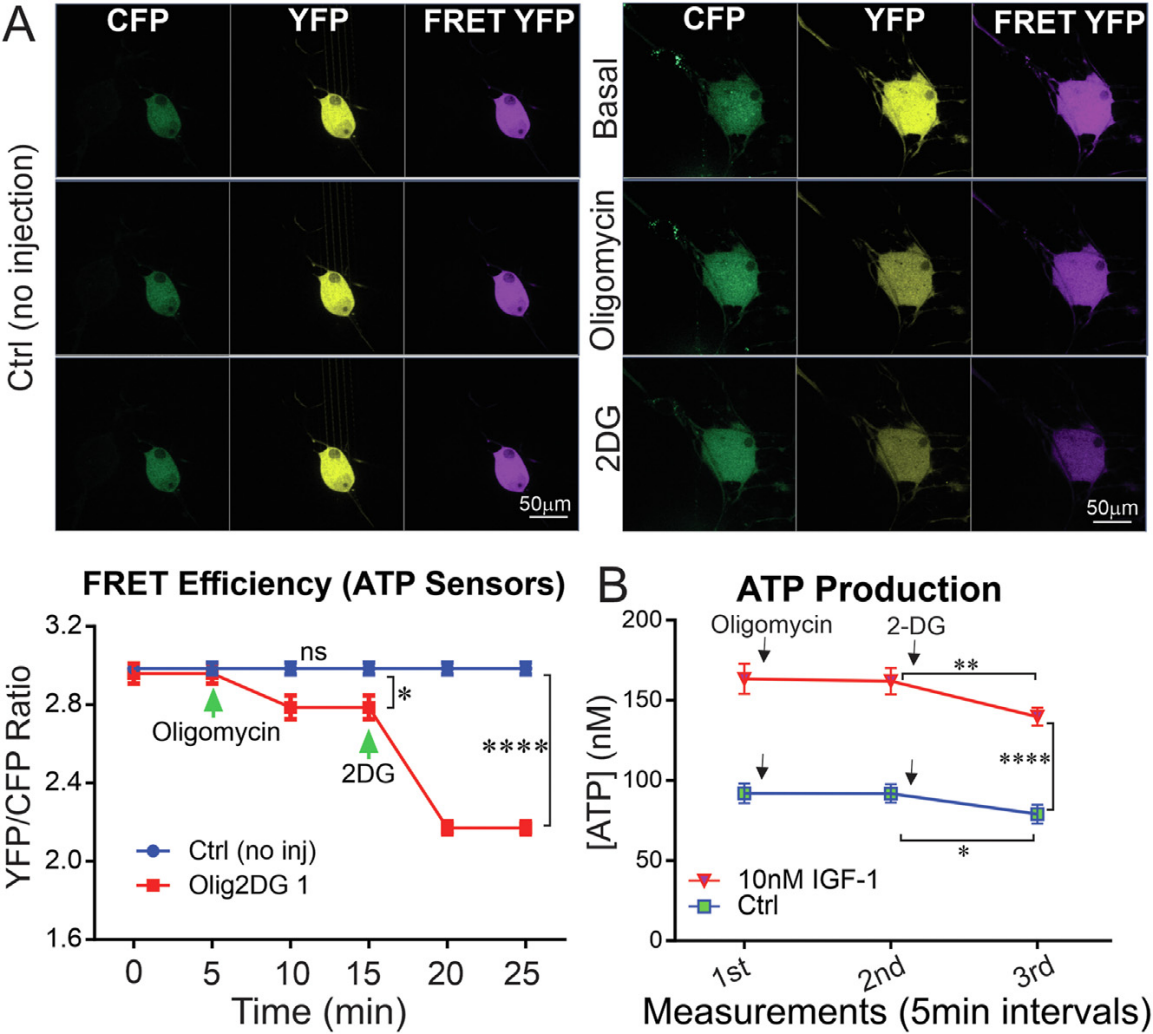
**Inhibition of glycolysis caused a greater decrease in ATP compared with inhibition of ATP synthase.** DRG neurons derived from adult control rats were transfected with YEMK (medium affinity) construct for 48 h. Images were taken using confocal microscope and FRET efficiency (YFP/CFP ratio) was calculated for each group of cultured neurons transfected with ATP sensors. (A) The group with no injection was considered as the control group. A group of cells were treated with oligomycin and then 2DG on-stage. FRET at basal level, after oligomycin (irreversible ATP synthase inhibitor) injection, and after 2-deoxy glucose (2-DG: glycolysis inhibitor) injection was measured. In (B), a similar approach was used to measure ATP levels using a luminescent-based ATP assay kit except that all treatments were done prior to one-time measurement of ATP. A subgroup of neurons was treated with 10 nM IGF-1 the day before the assay. In (A), data are mean ± SEM of N > 15 trials (at least 15 neurons from 15 distinct wells were analysed per group). In (B), data are mean ± SEM of N = 5 (5 wells per group); ∗ = p < 0.05 or ∗∗ = p < 0.01 or ∗∗∗∗ = p < 0.0001; analysed by one-way ANOVA with Dunnett's post-hoc test.

To delineate the contribution of the mitochondrial respiratory or glycolytic pathways to the production of ATP in sensory neurons, DRG neurons derived from adult control rats were transfected with YEMK (medium affinity) construct for 48 h and treated with specific inhibitors. Short-term treatment with oligomycin (irreversible and specific inhibitor of ATP synthase in mitochondria) significantly decreased FRET efficiency (ATP levels) in cultured DRG neurons (Figure 4A). The level of ATP in live cells was drastically diminished after the addition of 2-DG (an inhibitor of glycolysis) to the medium (Figure 4A).

In an independent experiment using an ATP assay kit, IGF-1-treated DRG neurons showed higher ATP levels after 24 h when compared to untreated DRG neurons. In this case, oligomycin did not acutely affect overall ATP levels in cultured DRG neurons compared to basal levels (Figure 4B). However, ATP levels were significantly lower (P < 0.05) in 2DG-treated DRG neurons when compared to untreated DRG neurons (Figure 4B). There was a 0.6 unit decrease in FRET efficiency after injection of 2DG (from 2.8 to 2.2 units), which accounts for almost 30% of total (2 units) FRET efficiency in cell bodies of sensory neurons. However, this drop was less than 30% (10–20%) of the absolute ATP measurement. These two complementary experiments imply that glycolysis can contribute up to 30% of ATP generation in sensory neurons even though the FRET signal and ATP concentrations do not exactly follow a linear relationship [40].

**Figure 5:**
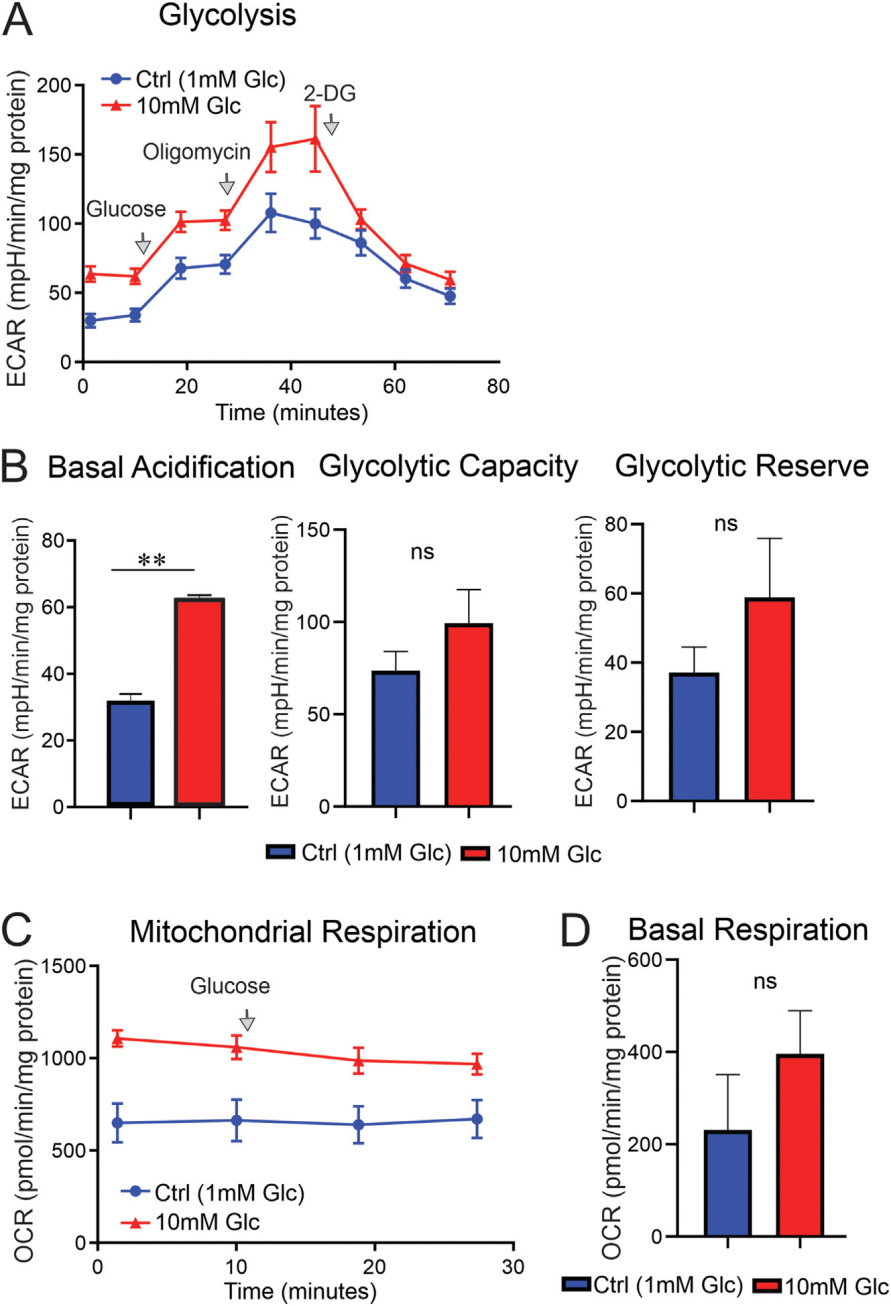
**Higher glucose concentration doubled basal glycolysis and basal mitochondrial respiration in cultured sensory neurons.** DRG neurons derived from adult control rats were cultured in the presence of 1 mM or 10 mM of glucose overnight and underwent glycolysis analysis and mitochondrial OCR assay using Seahorse XF24 analyser. All culture groups were starved of glucose for 1.5 h prior to the injection of 10 mM glucose programmed by the Seahorse analyser. More details on parameters of glycolysis and mitochondrial respiration are given in method section. In (A), glucose (10 mM), oligomycin (1 μM) and 2-deoxy-glucose (2DG: a glucose analogue) (50 mM) were sequentially injected to determine the extracellular acidification rate (ECAR). In (C), 10 mM glucose was injected to measure mitochondrial OCR under acute change in glucose concentration. In (A and C), first measurements of ECAR and OCR are considered basal levels of glycolysis and mitochondrial respiration, respectively. Basal glycolysis is not tabulated. Data are mean ± SEM of N = 4–5 replicates; analysed by Student's t-test.

To further scrutinise the role of mitochondria and glycolysis in provision of ATP, sensory neurons were cultured in the presence of very low glucose (1 mM) or normal glucose (10 mM) for 24 h. One millimolar glucose was used to provide suboptimal energy provision for DRG neurons to support normal glycolysis and mitochondrial respiration while not inducing cell death, and 10 mM was a normal physiological concentration of glucose for DRG neurons. Cells were starved 1.5 h (1 h incubation in new media and 30 min calibration and equilibration of the plate in the Seahorse bioanalyser) prior to the injection of 10 mM of glucose to all culture groups while inserted in the Seahorse bioanalyser. Both basal acidification and basal mitochondrial respiration were approximately 2-fold higher, in DRG neurons cultured in a medium containing 10 mM of glucose vs DRG neurons cultured in a medium containing 1 mM of glucose (Figure 5A–D). There was also more than a 1.5-fold increase in glycolytic reserve, although not statistically significant (Figure 5B). This component of the results indicates a major role of glucose as a source of energy for sensory neurons under basal conditions and demonstrates the significant effect of a drop in glucose concentration from 10 mM to 1 mM on cellular bioenergetics.

**Figure 6:**
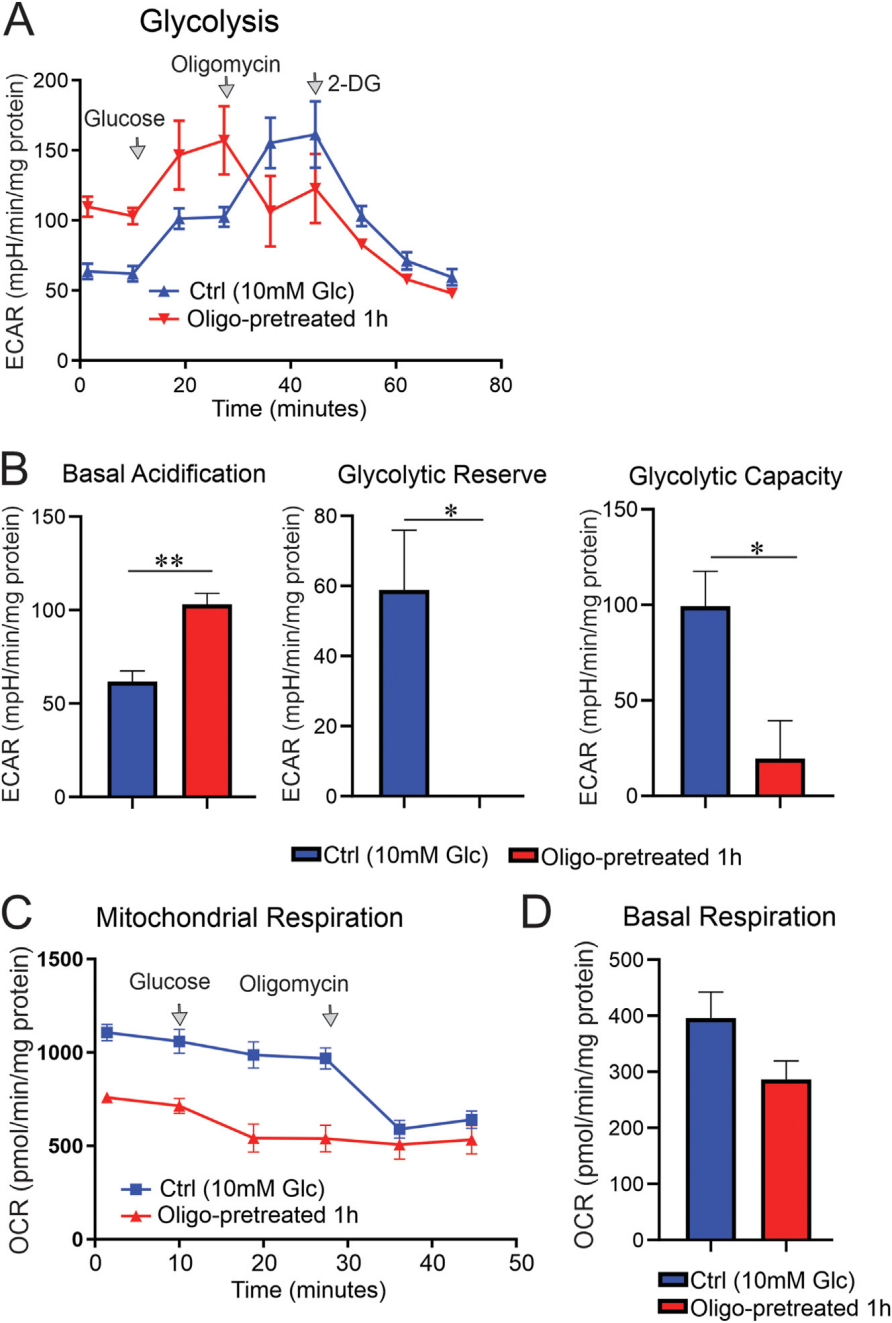
**Oligomycin treatment doubled glycolysis while decreasing mitochondrial respiration in cultured sensory neurons.** DRG neurons derived from adult control rats were cultured in the presence of 10 mM of glucose and incubated overnight. On the day of glycolysis analysis and mitochondrial OCR assay, culture groups were starved of glucose for 1.5 h prior to the injection of 10 mM glucose programmed by the Seahorse bioanalyser. A subgroup of neurons was pre-treated with 1 μM oligomycin for a total of 1 h before the measurements. In (A), glucose (10 mM), oligomycin (1 μM) and 2-deoxy-glucose (2DG: a glucose analogue) (50 mM) were sequentially injected to determine the extracellular acidification rate. In (C), glucose (10 mM) and oligomycin (1 μM) were sequentially injected and mitochondrial OCR was measured using Seahorse XF24 analyser. More details on parameters of glycolysis and mitochondrial respiration are given in the method section. Data are mean ± SEM of N = 4–5 replicates; ∗ = p < 0.05 or ∗∗ = p < 0.01; analysed by Student's t-test.

Control sensory neurons were cultured overnight in 10 mM of glucose, and pre-treatment with 1 μM of oligomycin (for 1 h) revealed that basal acidification doubled while mitochondrial respiration diminished by 1.8-fold compared to that in untreated cultured DRG neurons (Figure 6A–D). This suggests that glycolysis is the major source of energy under a 10-mM glucose concentration when there is compromised mitochondrial respiration (in this case, induced by oligomycin). Acute addition of glucose, however, further increased the glycolytic measurements in both oligomycin-treated and control groups by 1.5-fold (statistically not significant) without any major impact on mitochondrial respiration (Figure 6A–D). This increase was observed in the presence of significant reduction in glycolytic capacity and depletion of glycolytic reserve in the oligomycin-pretreated culture group (Figure 6B), indicating a negative feedback of mitochondrial dysfunction on glycolytic activity in the presence of 10 mM of glucose.

**Figure 7:**
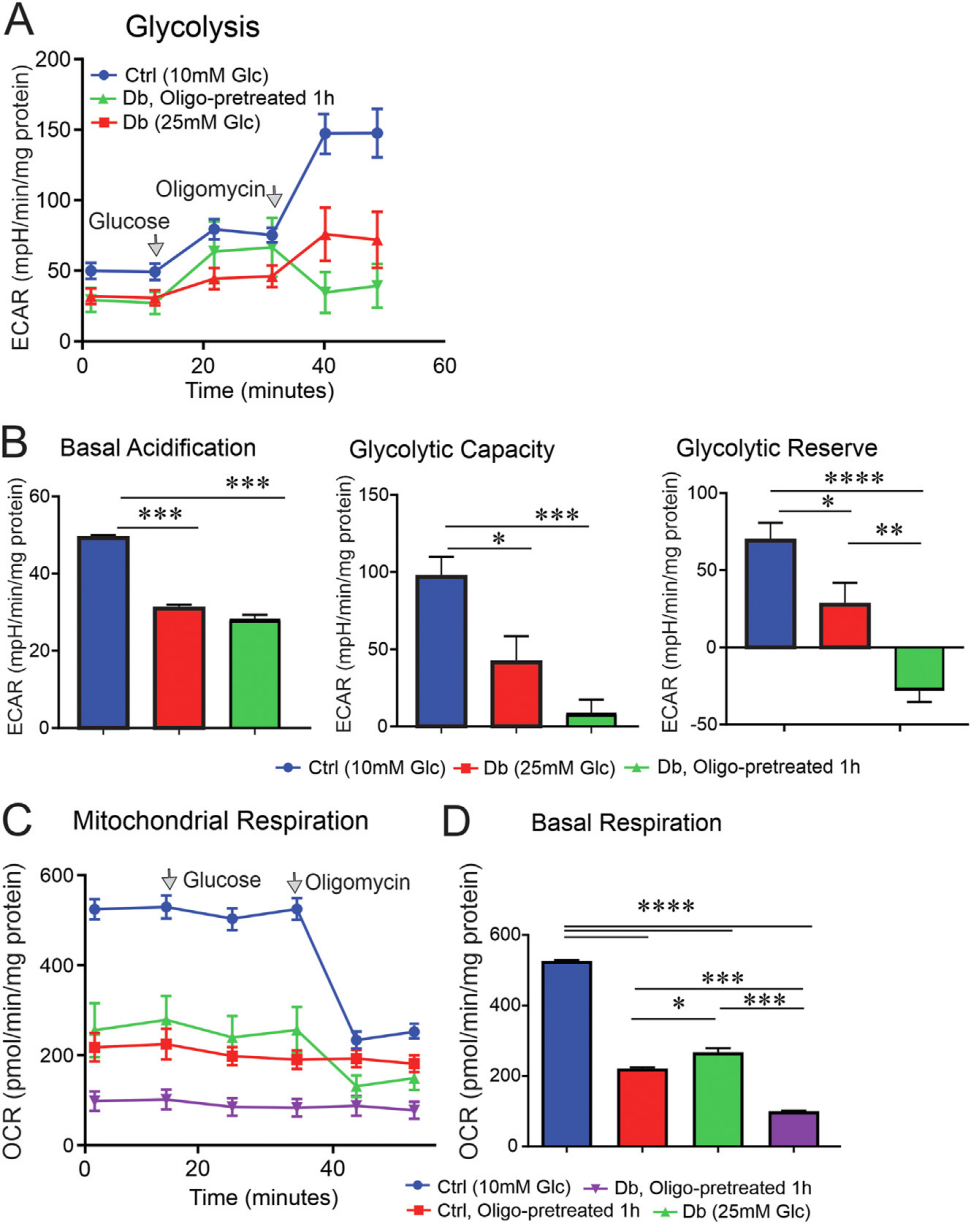
**DRG neurons from diabetic rats showed a deficit in glycolysis, and oligomycin treatment exacerbated the defect.** DRG neurons derived from control and diabetic rats were cultured in the presence of 10 mM or 25 mM glucose, respectively and incubated overnight. On the day of glycolysis analysis and mitochondrial OCR assay, all culture groups were starved of glucose for 1.5 h prior to the injection of 10 mM of glucose programmed by the Seahorse analyser. A subgroup of neurons was pre-treated with 1 μM of oligomycin for a total of 1 h before the measurements. In (A), glucose (10 mM) and oligomycin (1 μM) were sequentially injected to determine the extracellular acidification rate. The glycolytic capacity and reserve calculated in (B) are not corrected to basal levels since 2DG was not used as the last injection. In (C), glucose (10 mM) and oligomycin (1 μM) were sequentially injected and mitochondrial OCR was measured using Seahorse XF24 analyser. More details on parameters of glycolysis and mitochondrial respiration are given in the method section. Data are mean ± SEM of N = 5 replicates; ∗ = p < 0.05 or ∗∗ = p < 0.01; or ∗∗∗ = p < 0.001; or ∗∗∗∗ = p < 0.0001; analysed by one-way ANOVA with Tukey's post-hoc test.

We then cultured sensory neurons derived from control or diabetic rats overnight in the presence of 10 mM or 25 mM of glucose, respectively. All the culture groups were starved for 1.5 h prior to the injection of 10 mM of glucose while inserted in the Seahorse bioanalyser, then glycolytic measurements and mitochondrial respiration parameters were analysed. Basal acidification, glycolytic capacity, glycolytic reserve and mitochondrial basal respiration were significantly (P < 0.01) depressed in sensory neurons derived from diabetic rats when compared to sensory neurons from control rats (Figure 7A–D). In line with our control condition, pre-treatment of oligomycin depleted mitochondrial respiration and glycolytic reserve in sensory neurons derived from diabetic rats (Figure 7A–D).

## 4 Discussion

We demonstrate in the present study that ATP levels are measurable in live sensory neurons using ATP biosensors developed based on FRET technology. We found that there was a loss of ATP and energy supply in the neurites and cell bodies of DRG neurons derived from diabetic rats which were restored by IGF-1 treatment *in vitro*. Axonal area (longest neurite) displayed homogenous ATP levels along the neurite, while there was a lower level of ATP in the neurites compared to cell bodies of sensory neurons. We demonstrate for the first time a clear functional deficit in multiple parameters of glycolytic activity in DRG neurons derived from diabetic rats which was restored by IGF-1 treatment. Finally, we propose that glycolysis remains a significant source of energy for sensory neurons even in the absence of fully functional mitochondria (as is the case under oligomycin treatment or diabetic conditions).

There is a growing array of genetically encoded ATP biosensors developed as tools to perform real-time monitoring of the energy homeostasis in live cells [36,[41], [42], [43], [44], [45]]. All ATeam (AT) biosensors were first developed by Imamura et al. [36] and demonstrate different level of affinities to ATP (no affinity to ADP, GTP and dATP). These constructs are sensitive to pH or temperature change and are responsive to inhibition of glycolysis and oxidative phosphorylation. In our hands, with the exception the MGK construct, the low-medium affinity ATP biosensors showed robust signal, despite no detectable FRET signal for the mutant construct in cultured adult DRG neurons. To the best of our knowledge, we are the first group to use these FRET-based ATP biosensors to study bioenergetics in cultured sensory neurons derived from normal or diabetic rats under normoglycemic or hyperglycaemic conditions, respectively.

Mitochondria produce the majority of ATP required in cells; however, these organelles are structurally and functionally impaired in diabetic neuropathy, which could potentially affect ATP levels in DRG neurons. In individuals with diabetic neuropathy, mitochondria accumulated in axonal swellings of IENF [46,47]. Accumulation of fragmented mitochondria, possibly indicative of elevated fission, in autonomic ganglia and dorsal root has been demonstrated in STZ-diabetic and *db/db* mice, respectively [48,49]. Our laboratory and other groups have shown mitochondrial inner membrane depolarisation and reduced complex activities in sensory neurons in rodent models of type 1 and type 2 diabetes [[20], [21], [22],^33^,^50^]. Mitochondrial oxygen consumption rate in isolated DRGs from STZ-diabetic rats revealed a significant reduction compared to controls, but this required 4–5 months of type 1 diabetes to develop [21]. It has been proposed that suppression of the AMPK/peroxisome proliferator-activated receptor γ coactivator-1α (PGC-1α) axis, a master regulator of mitochondrial biogenesis, induced by hyperglycaemia triggers mitochondrial dysfunction in DRG neurons [51]. Additionally, alterations in glycolysis have been reported in diabetic neuropathy. For instance, pyruvate dehydrogenase kinase 2 (PDK2) and 4 (PDK4), key enzymes inhibiting pyruvate dehydrogenase, were increased in DRG tissue from STZ-diabetic mice. Furthermore, PDK2/4 knockout mice resisted pain hypersensitivity, macrophage infiltration, satellite cell activation and loss of peripheral nerve fibres after induction of type 1 diabetes [52]. Three months of diabetes suppressed hexokinase I activity and expression in lumbar DRG tissue in STZ-diabetic rats [31]. In a metabolomic/proteomic analysis, multiple glycolytic proteins were increased in the sciatic nerve but not in DRG tissue from STZ-diabetic rats after 12 weeks of diabetes; however, glycolytic intermediates, including glucose-6-phosphate, fructose 1,6-bisphosphate and glyceraldehyde-3-phosphate, did not significantly change [27]. Transcriptomic studies by Sas et al. confirmed the increase in expression of multiple components of glycolysis and oxidative phosphorylation in the sciatic nerves of 24-wk db*/db* mice [29]. In the former study, and confirming a previous report [28], metabolomic studies revealed significant deficits in a variety of glycolytic intermediates, including glucose-6-phosphate, phosphoenolpyruvate and 3-phosphoglycerate. In addition, TCA cycle intermediates citrate and isocitrate were depleted (in sural nerve, sciatic nerve and DRG) in *db/db* mice [28,29]. Most importantly, the report of Sas et al. used metabolic flux analysis to fully characterise metabolism in the nerve of *db/db* mice [29]. The findings were complex but revealed that metabolic flux from glucose and palmitate was impaired at an undetermined step, *e.g.,* contributing to reduced citrate, glutamate and succinate. Interestingly, flux from pyruvate was not impaired, and was actually enhanced in diabetic tissue. To summarise, these previous papers describe a deficit in nerve metabolism linked to impaired glycolysis and impaired use of fatty acid as an energy source in *db/db* mice [29]. This suppression of energy production is accompanied by an increase in expression of components of these pathways, possibly as a feedback mechanism to overcome the deficit in bioenergetics.

The higher glucose concentration (45 mM) decreased ATP/ADP levels and mitochondrial membrane potential and induced programmed cell death in cultured embryonic DRG neurons [53]. In the current study, we used functional measurements to reveal an energy deficit in the form of decreased ATP associated with suppressed glycolytic reserve and capacity in sensory neurons derived from diabetic rats using a Seahorse XF24-based analysis of glycolysis in live cells (a glycolysis stress test). The depressed glycolytic-dependent acidification observed in cultured DRG neurons from diabetic rats (Figure 6) could be due to decreased activity of glycolytic enzymes, including glucose-6-phosphate dehydrogenase and pyruvate dehydrogenase, and TCA cycle pathway components [37]. Another contributor could be impaired glucose uptake; however, we can only speculate at this juncture. These findings confirm the metabolomic and metabolic flux measurements of Sas et al. and Hinder et al. in nerves of *db/db* mice in which there were multiple deficits in the glycolytic pathway and its intermediates [28,29]. The functional findings of the current study and the descriptive findings of Sas et al. oppose the Brownlee hypothesis as it argued that hyperglycaemia increases glucose flux through glycolysis and the Krebs cycle, thus subsequently saturating the proximal axis of the electron transport system in mitochondria and driving up ROS levels [54,55]. However, supporting evidence for this hypothesis stemmed mainly from endothelial cell culture studies. Based upon our current work and that of other groups in DRG neurons or nerve tissue in diabetes, this theory must be re-appraised as the central idea does not support the functional and metabolic alterations actually being measured.

Metabolically, mitochondria are tightly dependent on glycolysis, and this linkage is highlighted by the presence of hexokinase I binding to the mitochondrial outer membrane [56,57]. Mitochondria are mostly generated and refurbished in the neuronal perikarya and have a higher density in the cell body than axons [[58], [59], [60]]. They are distributed equally along the axons, excluding the axonal tips [56]. Mitochondria are found concentrated in the axonal terminals to meet metabolic demand and protect the most vulnerable region of the DRG neuron against insulting conditions, such as diabetes. Defective fusion and increased fission of mitochondria in DRG neurites have been proposed as a mechanism driving mitochondrial fragmentation and impaired energy production in diabetes [49,61]. This is in line with our finding of higher ATP levels in the cell bodies compared with the neurites of sensory neurons, both of which were higher than their counterparts in DRG neurons from diabetic rats. In addition, the lower ATP levels in the axonal terminals could contribute to the cognitive impairment observed in animal models of diabetic neuropathy [62]. We also discovered that IGF-1 treatment (for 24 h) could reinstate the energy homeostasis in cultured DRG neurons, which is in line with IGF-1 enhancement of mitochondrial respiration and correction of intermediates of glucose metabolism in the diabetic condition [33]. This finding highlights the role of IGF-1 in accelerating glucose flux through glycolysis and mitochondrial respiration. To fulfil this role, IGF-1 binds to its receptor (IGF-1R) and mobilises insulin receptor substrate 2 (IRS-2) to phosphorylate and stimulate the Akt pathway [63]. Akt signalling inhibits glycogen synthase kinase-3β (GSK-3β) to promote glucose uptake and aerobic glycolysis in mammary tumour cells [63]. A metabolic flux analysis on mammary gland tumours from p53^R270H/+^WAP-Cre mouse revealed that activation of IGF-1R was associated with upregulated glycolysis [64]. In the Calu-1 cell line, IGF1 induced an increased nuclear localisation of PKM2 which correlated with an increased GLUT1 (glucose transporter) expression and glycolysis [65]. Elevated glycolytic (pyruvate kinase M1 and M2) and mitochondrial (MTCO2) enzymes improved glucose flux and were protective against chronic kidney disease in type 1 and type 2 diabetic patients [66].

Our data suggest that glycolysis (aerobic and anaerobic) makes up around 30% of the energy source for sensory neurons *in vitro* which is higher than the 10–12% reported for adult brain neurons. We also showed both acute mitochondrial dysfunction and excessive glucose supply triggers glycolysis (depletion of glycolytic reserve) to possibly compensate for the lack of energy supply in sensory neurons. The long-term diabetic condition most likely suppresses energy supplied via aerobic glycolysis due to a defect in glucose flux secondary to mitochondrial dysfunction. The significant upregulation of metabolic flux from pyruvate to lactate, an end-product of anaerobic glycolysis, in various tissues, including the kidney cortex, sciatic nerve and retina as early as 12 weeks of diabetes in *db*/*db* mice supports the generation of lactate substituting as a source of energy in case of defective aerobic glycolysis [29]. Metabolomic analysis of nerve tissue of 24-wk db*/db* mice actually revealed reduced lactate levels suggesting rapid conversion back to pyruvate or transport out of the nerve (most likely from Schwann cells) [28,29]. The deficit in glycolysis in the diabetic condition is further supported by our observation that addition of excessive glucose to the oligomycin-treated DRG neurons negatively impacted glycolytic reserve reflective of negative feedback of mitochondrial dysfunction in a chronic diabetic condition. Further investigations are required to dissect the precise share of aerobic glycolysis, oxidative phosphorylation and anaerobic glycolysis in the energy supply of neurons *in vivo* and the role each plays in protection against diseases, particularly diabetic neuropathy.

## 5 Conclusions

In conclusion, there is a paucity of ATP in the cell bodies and axons of sensory neurons derived from type 1 diabetic rats. The deficit in energy supply is predominantly caused by disturbed glycolysis accompanied by loss of mitochondrial function revealed through a functional analysis approach. IGF-1 acts on both energy sources to reverse the loss of energy supply in sensory neurons from diabetic rats and can be a potential therapy for the correction of bioenergetics in similar conditions.

## Funding

PF and DG were supported by a collaborative award from St Boniface Research and 10.13039/501100005005Ben-Gurion University. LK is supported by the Canadian Institute for Health Research Foundation Grant.

## Author contributions

PF and DG obtained funding for the work. PF, DG and MRA conceptualised the experiments. PF and MRA co-wrote the first version of the manuscript. MRA designed and performed all the experiments. DS generated and maintained type 1 diabetic rats and helped with the Seahorse assay. VM and LK aided in setting up and performing the FRET measurements. DG and LK edited the manuscript.
